# Tuberculosis Incidence and Predictors Among People Living With HIV After Isoniazid–Rifapentine (3HP) Preventive Therapy in Ethiopia: A Retrospective Cohort Study

**DOI:** 10.1155/jotm/4521687

**Published:** 2026-10-06

**Authors:** Edao Sinba Etu, Edao Wajiso Kallo, Kelil Husen Lenbo, Biftu Geda, Kemal Ahmed Kuti, Musa Kumbi Ketero, Habtemu Jarso Hebo, Shumi Negewo Berarti, Mark Spigt

**Affiliations:** ^1^ Department of Public Health, School of Health Science Shashemene Campus Madda Walabu University Shashemene Ethiopia mwu.edu.et; ^2^ Department of Family Medicine Faculty of Health Medicine and Life Science Care and Public Health Research Institute Maastricht the Netherlands; ^3^ Melka Oda General Hospital Oromia Regional Health Bureau Shashemene Ethiopia

**Keywords:** 3HP, Ethiopia, incidence, risk factors, tuberculosis, West Arsi

## Abstract

**Background:**

Global reports of tuberculosis (TB) in 2024 estimated 10.7 million cases, of which 5.8% were HIV‐positive individuals. Despite the implementation of TB preventive therapy, TB incidence rates remain high, especially among HIV‐infected patients.

**Methods:**

A retrospective cohort study was conducted from 1 October 2020 to 31 October 2025. A two‐stage sampling procedure was used to select both health facilities and study participants. First, seven public health facilities in the West Arsi zone were selected using simple random sampling. Second, 426 people living with HIV were randomly selected from ART registers of the selected health facilities. The data were collected via the Kobo Toolbox and analysed via SPSS Version 26. Kaplan‒Meier estimates were used for time‒event distribution; for survival, curves were compared via log‐rank tests. Risk factors for active TB were identified through Cox proportional hazards regression, with significance at *p* < 0.05.

**Results:**

A total of 426 HIV‐positive patients were followed for 1867 person‐years, during which 71 individuals (16.7% of participants) developed TB, resulting in a TB incidence density rate of 3.78 per 100 person‐years. The identified risk factors for developing active TB were WHO Clinical Stages II to IV (adjusted hazard ratios [AHR] = 3.44 [95% CI: 1.50–7.85], 5.41 [95% CI: 2.47–11.84] and 7.52 [95% CI: 3.09–18.32], respectively). Participants who received daily isoniazid for 6 months (INH/6H) and those who did not receive any TPT regimen had significantly greater hazard of developing TB compared to those receiving 3HP (AHR = 5.01, 95% CI: 2.35–10.65 and AHR = 12.53, 95% CI: 5.65–27.79, respectively). Similarly, individuals with haemoglobin (HBg) levels < 11 g/dL had a markedly increased risk of TB (AHR = 2.46, 95% CI: 1.31, 4.60), as did those with a body mass index < 18.5 kg/m^2^ (AHR = 4.37, 95% CI: 2.14, 8.95).

**Conclusion:**

This study revealed that 16.7% of participants developed active TB. Significant risk factors for TB incidence among HIV‐positive individuals were identified, highlighting the need for targeted preventive strategies to address these risks, particularly in high‐risk populations.

## 1. Introduction

Tuberculosis (TB) is preventable and, in most cases, curable if treated properly [1]. TB is a contagious illness caused by a bacterium called *Mycobacterium tuberculosis*, which is transmitted through airborne particles when individuals with TB cough, sneeze or sing. Not everyone infected with TB becomes sick. There are two TB‐related conditions: latent TB infection (LTBI) (or inactive TB) and TB disease [2]. Without proper interventions, TB can lead to severe illness and death.

TB remains a major public health crisis that significantly affects global health. A total of 10.7 million people developed TB in 2024 [3], decreased from 10.8 million in 2023 [4], for the first time since 2020, following 3 consecutive years of increases (2021–2023). People living with HIV (PLHIV) account for an estimated 5.8% (620,600) of all incident cases (10.7 million) of TB in 2024 (3). PLHIV are 18 times more likely to develop TB than those without it. The coinfection epidemic poses an urgent challenge, especially in sub‐Saharan Africa, where the burden is disproportionately high [5, 6].

Ending the global TB epidemic remains a distant goal, despite efforts to reduce TB incidence, with a target of a 90% reduction in new TB cases by 2035 [6]. Globally, the net TB incidence rate reduction from 2015–2024 was 23%, which is slightly higher than the 8.3% reduction achieved from 2015–2023. The incidence rate reduction is far from the World Health Organization (WHO) End TB Strategy milestone of a 75% reduction in TB incidence by 2025 [4]. The global TB incidence rate (new cases per 100,000 people per year) is estimated to have increased by 4.6% between 2020 and 2023, from 129 in 2020 to 134 in 2023, due to the COVID‐19 pandemic impact [6]. The African and European regions have made significant progress, with reductions of 42% and 38%, respectively [4]. Although progress has been made, Ethiopia still faces challenges with high rates of coinfection [5, 6]. According to the Ethiopian National TB and HIV Programmatic Management of LTBI Guideline, 3 months of weekly isoniazid plus rifapentine (3HP) is the preferred TB preventive therapy (TPT) regimen for all PLHIV aged 15 years and older who are not receiving a protease inhibitor (PI)– or nevirapine (NVP)–based antiretroviral regimen and have no contraindications to 3HP. Six months of isoniazid preventive therapy (IPT) is recommended for children, adolescents and adults living with HIV who are receiving a PI‐based antiretroviral therapy (ART) regimen or have other contraindications to 3HP [7].

In Ethiopia, longitudinal studies from regions such as Northwest [8, 9], Northeast [10], Central [11–14] and Southwest [15, 16] have examined the incidence of TB among PLHIV prior to the national implementation of 3HP. Furthermore, these findings have been inconsistent, making it challenging for program managers, policymakers, healthcare providers and researchers to design effective, context‐specific interventions to address TB–HIV coinfection and contribute to global TB elimination efforts.

However, the strength of our study lies in its use of comprehensive longitudinal data collected after the initiation of 3HP in Ethiopia. To our knowledge, evidence on regimen‐specific TB incidence and its predictors following the implementation of the 3HP regimen in routine HIV care in Ethiopia remains limited. By addressing this gap, our study not only contributes to the national understanding of TB–HIV dynamics but also contributes valuable data to the broader global literature.

## 2. Materials and Methods

### 2.1. Study Area, Study Period and Study Design

Shashemene, the capital of the West Arsi Zone, is located approximately 250 km south of Addis Ababa. In this zone, there is one referral hospital, three general hospitals, three primary hospitals and 90 health centres. Five hospitals and 20 health centres currently provide ART services to a total of 8557 patients. When individuals test positive for HIV, they are enrolled in an ART clinic for comprehensive HIV care. A multidisciplinary team that includes physicians, nurses, public health professionals, laboratory technologists, pharmacists and data clerks supports the clinic. To diagnose TB in HIV‐positive patients, the ART clinic employs various methods, such as acid‐fast bacilli (AFB) testing, chest X‐rays, urine lipoarabinomannan (LAM) tests and the GeneXpert MTB/RIF assay. All HIV‐infected adult patients received ART treatment, regardless of their CD4 count or WHO clinical stage.

An institution‐based retrospective cohort study was conducted among HIV patients enrolled at selected health facilities (HFs) in the West Arsi Zone of Southern Ethiopia. Data were extracted between 1 December 2025 and 31 January 2026.

### 2.2. Study Population and Eligibility Criteria

HIV‐positive individuals were newly registered at the ART clinic between 1 October 2020 and 31 October 2021, following the national implementation of 3HP. Randomly selected PLHIV aged 18 years and above who fulfilled the inclusion criteria were followed retrospectively for up to 5 years (until 31 October 2025). Individuals with incomplete records, patients not receiving ART and patients with a known active TB or LTBI at the time of recruitment were excluded from the study.

### 2.3. Sample Size Determination

For the first objective, a sample size was calculated via a single proportion formula considering a proportion of 21.4% of patients with TB among HIV‐positive patients in Arba Minch, Ethiopia [17], a 95% level of confidence, a degree of precision of 5% and an assumed loss to follow‐up of 10%. The total sample size was 426. The maximum sample size calculated for the second objective was 231 (Supporting Table 1), which is smaller than the sample size calculated for the first objective. Thus, the final sample size was 426. Furthermore, with 71 incident TB events observed during follow‐up, the final multivariable Cox regression model was refined to include a limited number of predictors in accordance with the recommended events‐per‐predictor‐variable (EPV) criterion to improve model stability, minimise the risk of overfitting and enhance the reliability of the estimated hazard ratios.

### 2.4. Sampling Procedure

A two‐stage sampling procedure was followed.

#### 2.4.1. Stage 1: Selection of HFs

This study was conducted in 7 public HFs randomly selected from 26 HFs found in the West Arsi Zone, which were selected as follows: We stratified the 26 public HFs by level (hospitals vs. health centres). We used a simple random sampling technique to determine the number of facilities sampled from hospitals and health centres. As a result, we selected three hospitals—Dodola General Hospital, Shashemene Comprehensive Specialized Hospital and Melak Oda General Hospital—and four health centres—Arsi Negele Health Center, Abosto Health Center, Adaba Health Center and Hasasa Health Center—from the six hospitals and 20 health centres that provide ART services in the zone (Figure 1).

**FIGURE 1:**
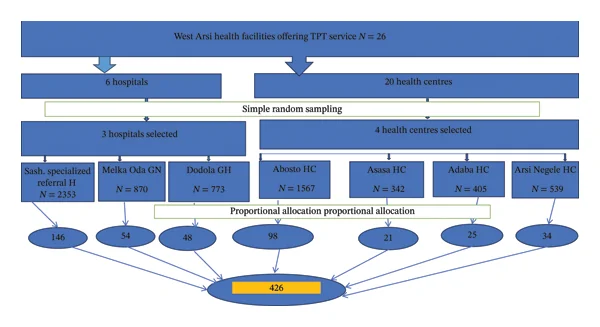
Schematic sampling of PLHIV and health facilities in West Arsi, Ethiopia, from 1 October 2020 to 31 October 2025.

#### 2.4.2. Stage 2: Selection of Study Participants

The study included a total of 426 individuals. The selection process involved sorting and reviewing the medical records of all HIV‐positive adults receiving ART between 1 October 2020 and 31 October 2021. To achieve the desired sample size, patient charts from a pool of 6849 adults living with HIV/AIDS in seven selected HFs were proportionally allocated across the specified HFs in the West Arsi. Patient charts were then selected using a simple random sampling method within each selected facility (Figure 1).

### 2.5. Measurement and Study Variables

The primary outcome variable of this study was the incidence of TB among HIV‐positive patients, calculated as the number of new TB cases divided by total person‐years (PYs) of follow‐up. For individuals who did not develop TB, the follow‐up period from 1 October 2020 to 31 October 2025 was classified as TB‐free. For individuals who developed TB, TB‐free survival time was calculated from the time of enrolment until the onset of TB. In this study, an event was considered any form of TB that was diagnosed clinically or radiographically and confirmed by laboratory examinations (sputum smear, GeneXpert, culture, urine LAM).

Patients who were receiving anti‐TB treatment at the time of enrolment were excluded from the study. HIV‐positive individuals who were lost to follow‐up, transferred to another facility, died or were not diagnosed with TB by the end of the follow‐up period were classified as censored. The study examined various variables, including age, sex, educational background, employment status, residence, religion, family size and marital status. Clinical characteristics such as WHO clinical stage, baseline CD4 count, bedridden functional status, TB history, body mass index (BMI) and haemoglobin (HBg) levels, as well as sociodemographic and economic factors, were also assessed.

### 2.6. Data Collection Procedure, Data Collection Tools and Data Quality Control

This study used retrospective data from patient records monitored during routine service delivery. In Ethiopian public HFs, medical records for HIV‐infected patients are documented via standardised national formats and registers (S1: Annex A and B), which are routinely monitored for completeness and accuracy by facility‐level data clerks and supervisors as part of the national health information system. These records include key clinical indicators, treatment adherence and TB screening outcomes and are periodically reviewed during supervision and reporting cycles to ensure data quality.

For this study, data were extracted using a pretested form via the Kobo Toolbox, with a pilot version tested at Kokosa General Hospital, and adjustments were made to improve clarity and alignment with medical record formats before they were finalised for chart review. Eight trained data clerks extracted information from the patient records and registration books of HIV‐infected patients followed up between 1 October 2020 and 31 October 2025, under supervision to ensure the accuracy, consistency and completeness of the collected data. To ensure data quality, data quality assurance procedures were established before commencement of data extraction. In all selected HFs, patient intake and follow‐up information were systematically documented in SmartCare (electronic medical record/data management system used in Ethiopia to record and manage patient‐level health information) using standardised formats. These routinely maintained records served as the primary source of information for the present study. During data extraction, an electronic Kobo Toolbox data collection form was designed with mandatory response fields to minimise missing data. The principal investigator reviewed the extracted data daily, resolving any inconsistencies or discrepancies directly with data clerks. Following data cleaning and verification, all 426 records originally selected through the random sampling procedure had complete information for all variables included in the analysis. No records were excluded or replaced because of missing information, and no additional records were sampled to achieve the final sample size of 426. Finally, records were thoroughly cleaned prior to analysis, and only those with complete information were included in the final analysis.

### 2.7. Data Processing and Analysis

Data were extracted via a data extraction form and exported to SPSS Version 26 for cleaning, checking for completeness and analysis. The patients’ follow‐up characteristics are presented as medians, interquartile ranges and frequency distributions. The incidence density of TB was calculated per 100 PYs. Time‐to‐event distributions were estimated via Kaplan‒Meier estimates. A separate graph of Kaplan‒Meier survival functions and log‐rank tests was generated for each categorical variable to compare survival between different exposure groups. Cox proportional hazards regression model was used to screen variables for the final model. Variables with a *p* value of ≤ 0.25 in the bivariate analysis were fitted into the multivariable Cox proportional hazards regression model. Multicollinearity was assessed using variance inflation factor (VIF), tolerance statistics, eigenvalues, condition index and variance decomposition proportions. The following cutoff values were used to evaluate multicollinearity: VIF < 10, tolerance > 0.1, condition index < 30, eigenvalues not approaching zero and absence of clustered variance proportions greater than 0.5 under high condition indices. Variables exceeding these thresholds were considered to demonstrate problematic multicollinearity. The proportional hazards assumption was evaluated using Schoenfeld residual tests and log‐minus‐log survival plots. Neither the global test (*χ* ^2^ = 4.59, df = 11, *p* = 0.95) nor any individual covariate demonstrated evidence of violating the proportional hazards assumption (all covariate‐specific *p* > 0.05). Visual inspection of Schoenfeld residual plots revealed no systematic time‐dependent trends, while log‐minus‐log survival curves for each covariate were approximately parallel (Supporting Figures 2, 4, 6 and 8), indicating that the final Cox regression model was appropriate for the data. Finally, an adjusted hazard ratio (AHR) with its corresponding 95% confidence interval was reported to indicate the presence of a significant association between the explanatory and outcome variables, with a *p* value < 0.05 in the multivariate analysis. The model’s goodness of fit was assessed via the Cox–Snell residual plot. This plot calculates an empirical estimate of the cumulative hazard function on the basis of the Kaplan–Meier survival estimates, taking the Cox–Snell residuals as the time variable (S2: Supporting Figure 9). Because death precludes the subsequent occurrence of TB, a Fine and Gray competing risks regression model was performed as a sensitivity analysis to account for death as a competing event. Subdistribution hazard ratios (SHRs) with 95% confidence intervals were estimated to evaluate the association between predictors and incident TB while accounting for the competing risk of death. The findings from the Fine and Gray model were compared with those of the cause‐specific Cox proportional hazards model to assess the robustness of the results.

### 2.8. Operational Definition

An incident TB case was defined in this study as an event and was diagnosed via sputum smear positive (at least two samples), urine LAM, chest X‐ray (suggestive of TB findings), GeneXpert and culture positive during follow‐up, all of which were ascertained retrospectively.

TPT: In this study, TPT was defined, in accordance with national guidelines, as administration of isoniazid for at least 6 months or 3HP for 3 months.

Cumulative incidence of TB: This rate was calculated by dividing the total new occurrences of TB by the total number of sampled patients.

Incidence rate of TB: The incidence rate in this research was calculated by dividing all new occurrences of TB by the total follow‐up time of patients by year.

ART adherence level: According to the most common adherence classification system used in Ethiopia, adherence was categorised as good (missing ≤ 3 doses per month or ≥ 95%), fair (missing 4–8 doses per month or 85%–94%) or poor (missing ≥ 9 doses per month or < 85% adherence).

TB diagnosis: TB was diagnosed via microscopic examination of sputum smears, GeneXpert, chest radiology, fine‐needle aspiration of lymphadenopathy, urine LAM (especially for HIV‐infected patients), cytology with very high clinical suspicion and Mycobacterium culture [18].

LTBI: It refers to a persistent immune response to Mycobacterium TB antigens without any signs of active TB or illness [19]. Those with LTBI carry the TB bacteria in their body but remain asymptomatic as the bacteria are dormant. Clinical screening using symptom‐based criteria can safely identify individuals eligible for TPT—specifically, the presence of any one of the following symptoms: current cough, fever, unintentional weight loss or night sweats. The routine use of TST and IGRA, chest X‐ray or other TB diagnostic tests is not recommended for the diagnosis of LTBI in order to initiate TPT [20].

## 3. Results

### 3.1. Sociodemographic Characteristics of the Participants

Data extracted from the charts and registration books of 426 HIV‐positive patients were included in the analysis. Among these, 161 (37.8%) were in the age category of 25–34 years. More than half, 246 (57.7%), of the HIV‐infected patients were female, with a substantial majority, 291 (68.3%), residing in urban areas. Additionally, a significant number of patients, 364 (85.4%), disclosed their HIV status to family members (Table 1).

**TABLE 1:** Sociodemographic characteristics of HIV‐positive patients at selected health facilities in the West Arsi Zone, southern Ethiopia, from 1 October 2020 to 31 October 2025.

Variable		Frequency	%
Age of the patient	18–24	58	13.6
	25–34	161	37.8
	35–44	125	29.3
	≥ 45	82	19.2

Sex of the patient	Male	180	42.3
	Female	246	57.7

Religion of the patient	Protestant	75	17.6
	Orthodox	202	47.4
	Muslim	122	28.6
	Other	27	6.3

Residence	Urban	291	68.3
	Rural	135	31.7

Marital status of the patient	Single	69	16.2
	Married	210	49.3
	Widowed	37	8.7
	Divorced	110	25.8

Educational status of the patient	Unable to read and write	114	26.8
	Grade 1–6	137	32.2
	Grade 7–12	127	29.8
	Diploma and above	48	11.3

Disclosed HIV status	Disclosed	364	85.4
	Not disclosed	62	14.6

Occupation	Farmer	54	12.7
	Merchant	116	27.2
	Employed	115	27.0
	Other	141	33.1

Family size	≤ 5	392	92
	> 5	34	8.0

### 3.2. Baseline Clinical Characteristics

Of the participants, 259 (60.8%) were classified as WHO Clinical Stage I. 91.6% of the participants had a CD4 count ≥ 200 cells/mm^3^. The majority, 349 (81.9%), had a HBg level of ≥ 11 g/dL. One‐third (30.75%) of the participants had a BMI < 18.5 kg/m^2^. During the follow‐up period, 287 (67.4%) of the participants changed their initial regimen. Furthermore, the majority, 364 (85.4%), of the participants had a history of good treatment adherence, and 89.2% received TPT, of which 286 (67.1%) were receiving 3HP. BMI was calculated from the recorded weight and height measurements using the standard formula (kg/m^2^), with BMI < 18.5 kg/m^2^ classified as underweight. In contrast, nutritional status was extracted directly from the patients’ medical records, where it had been documented by clinicians based on routine clinical assessment. Consequently, the two variables were not expected to be completely concordant. This resulted in a difference of 15 participants between the number classified as having BMI below 18.5 kg/m^2^ (*n* = 131) and those recorded as having moderate or severe wasting (*n* = 116) (Table 2).

**TABLE 2:** Baseline clinical, laboratory, ART and other medication‐related information of HIV‐infected patients receiving HIV care at selected health facilities in the West Arsi Zone, southern Ethiopia, from 1 October 2020 to 31 October 2025.

Characters		Frequency	%
WHO clinical stage	I	259	60.8
	II	70	16.4
	III	72	16.9
	IV	25	5.9

Duration on ART	< 6 months	6	1.4
	≥ 6 months	420	98.6

Functional status of the patient	Working	360	84.5
	Nonworking^∗∗^	66	15.5

BMI	< 18.5	131	30.75
	≥ 18.5	295	69.25

Nutritional status	Normal	295	69.2
	Moderate wasting	114	26.76
	Severe wasting	17	3.99

HBg level	< 11	77	18.1
	≥ 11	349	81.9

TB preventive therapy was taken	YES	380	89.2
	NO	46	10.8

Cotrimoxazole preventive therapy taken	YES	258	60.6
	NO	168	39.4

TPT regimens received	3HP	286	67.1
	INH/6H	94	22.1
	Not received	46	10.8

Viral load	High	60	14.1
	Suppressed	209	49.1
	Not done	157	36.9

Regimen changed	YES	287	67.4
	NO	139	32.6

Reason for the regimen change	Side effect	48	11.3
	Tuberculosis	1	0.2
	Other	234	54.9
	No regimen change	143	33.6

CD4 count	< 200	36	8.4
	≥ 200	390	91.6%

Adherence to ART	Good	364	85.4
	Fair	51	12.0
	Poor	11	2.6

TB type recoded	PTB	57	13.4
	EPTB	14	3.3

^∗∗^Ambulatory or bedridden.

### 3.3. Incidence of TB

During the five‐year follow‐up, 426 participants contributed a total of 1867 PYs of observation. Throughout the study, 71 individuals (16.7% of participants) developed TB; five died, and the remaining 350 individuals were censored (transferred out, loss to follow‐up or TB‐negative at study end). The overall TB incidence rate was 3.78 cases per 100 PYs. Of 71 TB cases, 57 (80.3%) were pulmonary TB and 14 (19.7%) were extrapulmonary TB (Figure 2). TB incidence varied substantially by TPT regimen. Participants who received the 3HP regimen had the lowest TB incidence rate, at 0.75 cases per 100 PYs (95% CI: 0.41–1.40). In contrast, participants who received INH/6H had an incidence rate of 10.06 cases per 100 PYs (95% CI: 7.32–13.83), while those who did not receive TPT had the highest incidence rate, 13.24 cases per 100 PYs (95% CI: 8.80–19.93).

**FIGURE 2:**
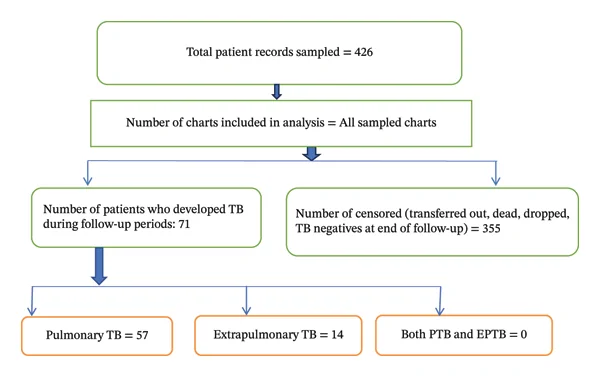
Incidence of tuberculosis and type of TB recorded among HIV patients in West Arsi, Ethiopia, from 1 October 2020 to 31 October 2025.

The mean survival time was 55.94 months. The cumulative probabilities of remaining TB‐free at the end of 1 year, 2 years, 3 years, 4 years and 5 years of follow‐up were 99%, 98%, 91%, 86% and 82%, respectively. The Kaplan–Meier survival analysis demonstrated a gradual decline in event‐free survival during the follow‐up period. At 60 months, the cumulative survival probability was approximately 82% (Figure 3).

**FIGURE 3:**
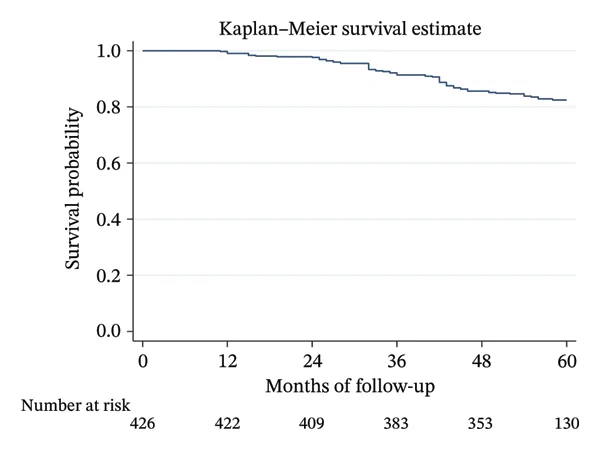
The overall Kaplan‒Meier survival curve of the TB‐free survival time of HIV‐positive adults on ART at selected health facilities in the West Arsi Zone, south‐eastern Ethiopia, from 1 October 2020 to 31 October 2025.

### 3.4. Predictors of TB in HIV‐Infected Patients

Eleven variables identified in the bivariate analysis were considered for inclusion in the multivariable Cox regression analysis model. These included marital status, functional status, BMI, nutritional status, TPT taken, past TB treatment history, TPT regimens, HBg level, WHO clinical stage, CD4 and medication adherence.

Before fitting the final model, we performed a comprehensive assessment of multicollinearity. Although moderate correlations remained between BMI and nutritional status and between TPT uptake and TPT regimen, these did not exceed accepted diagnostic thresholds for severe multicollinearity. Nevertheless, given the recommended rule of approximately 10–20 EPV for Cox regression models and the 71 TB events observed in this study, we further refined the model to improve parsimony, enhance model stability and reduce the risk of overfitting. Accordingly, nutritional status and TPT uptake were excluded because they were correlated with BMI and TPT regimen, respectively, while BMI and TPT regimen were considered more clinically informative predictors.

Following refinement of the multivariable model, multicollinearity was reassessed and no evidence of problematic multicollinearity remained. The proportional hazards assumption was then evaluated using covariate‐specific Schoenfeld residual tests. The results demonstrated that past TB treatment history (*p* = 0.006) and marital status (*p* = 0.02) violated the proportional hazards assumption. Because the Cox proportional hazards model assumes that hazard ratios remain constant over time, these variables were not appropriate for inclusion in the final model. Therefore, past TB treatment history and marital status were excluded from the final multivariable Cox regression model. The proportional hazards assumption was subsequently reassessed for the defined model, and neither the global test nor any of the remaining covariates demonstrated evidence of violation, supporting the validity of the final model.

The final multivariable Cox regression model therefore included seven predictors: functional status, BMI, TPT regimen, HBg level, WHO clinical stage, CD4 count and ART medication adherence. The final model demonstrated acceptable collinearity diagnostics, with all VIF values below 5, tolerance above 0.10 and no evidence of multicollinearity based on condition indices, eigenvalues and variance decompositions.

A multivariable Cox regression analysis indicated that WHO Clinical Stages II, III and IV, a BMI < 18.5 kg/m^2^, HBg levels < 11 g/dL and receiving INH/6H and not receiving any TPT regimen were significant predictors of TB incidence.

The findings revealed that WHO clinical stages advanced, particularly Stages II (AHR = 3.44; 95% CI: 1.50, 7.85; *p* = 0.003), III (AHR = 5.41; 95% CI: 2.47, 11.84; *p* < 0.01) and IV (AHR = 7.52; 95% CI: 3.09, 18.32; *p* < 0.001), indicating a strong correlation between disease severity and TB incidence. Individuals with a BMI < 18.5 kg/m^2^ had a significantly greater TB incidence (AHR = 4.37; 95% CI: 2.14, 8.95; *p* < 0.001), indicating that undernutrition may play a critical role. Patients with HBg level < 11 g/dL had a significantly increased risk of TB (AHR = 2.46; 95% CI: 1.31, 4.60; *p* = 0.005). Furthermore, the participants who received daily isoniazid for 6 months (INH/6H) and those who did not receive any TPT regimen had significantly higher risk of developing TB compared with those receiving 3HP (AHR = 5.01; 95% CI: 2.35, 10.65; *p* < 0.001; AHR = 12.53; 95% CI: 5.65, 27.79; *p* < 0.001, respectively) (Table 3).

**TABLE 3:** Bivariate and multivariate Cox regression analyses to identify predictors of tuberculosis among HIV‐positive adults on ART at the Public Health Facility in southern Ethiopia from 1 October 2020 to 31 October 2025.

Variables	Incidence of TB		Crude HR (95%CI)	*p* value	AHR (95% CI)	*p* value
	Yes	No				
	*n* (%)	*n* (%)				
TPT regimen received						
3HP	10 (3.50)	276 (96.5)	1	1	1	
INH/6H	38 (40.43)	56 (59.57)	14.24 (7.09, 28.61)	< 0.001	5.01 (2.35, 10.65)	< 0.001^∗^
Not received TPT	23 (50.00)	23 (50.00)	19.83 (9.42, 41.74)	< 0.001	12.53 (5.65, 27.79)	< 0.001^∗^
WHO clinical stage						
I	11 (4.2)	248 (95.8)	1		1	
II	15 (21.4)	55 (78.6)	5.68 (2.61, 12.38)	0.003	3.44 (1.50, 7.85)	< 0.001^∗^
III	27 (37.5)	45 (62.5)	11.33 (5.62, 22.88)	< 0.001	5.41 (2.47, 11.84)	< 0.001^∗^
IV	18 (72)	7 (28)	26.75 (12.60, 56.81)	< 0.001	7.52 (3.09, 18.32)	< 0.001^∗^
Functional status						
Working	37 (10.3)	323 (89.7)	1		1	1
Nonworking	34 (51.52)	32 (48.5)	6.26 (3.92,9.99)	0.07	1.23 (0.70,2.21)	0.45
BMI						
< 18.5	54 (41.54)	76 (58.5)	8.91 (5.16, 15.38)	< 0.001	4.37 (2.14, 8.95)	< 0.001^∗^
≥ 18.5	17 (5.74)	279 (94.3)	1		1	
Patient haemoglobin levels						
< 11	52 (14.9)	297 (85.1)	2.73 (1.02, 3.93)	0.04	2.46 (1.31, 4.60)	0.005^∗^
≥ 11	19 (24.7)	58 (75.3)	1		1	1
CD4						
< 200	12 (33.3)	24 (66. 7)	2.44 (1.31, 4.53)	0.005	1.71 (0.81, 3.62)	0.16
≥ 200	59 (15.13)	331 (84.9)	1		1	1
Adherence to ART						
Good	53 (14.6)	311 (85.4)	0.22 (0.09, 0.56)	0.001	0.44 (0.16,1.21)	0.11
Fair	13 (25.5)	38 (74.5)	0.41 (0.15, 1.16)	0.09	0.77 (0.26, 2.28)	0.64
Poor	5 (45.45)	6 (54.6)	1		1	

^∗^Statistically significant.

To assess the potential influence of death as a competing event, a Fine and Gray competing risks regression was performed. During follow‐up, 71 participants developed TB, five died before developing TB (competing events), and 350 were censored. The overall competing risks model was statistically significant (Wald *x* ^2^ = 210.07, *p* < 0.001). The results were consistent with those of the Cox proportional hazards model. Compared with participants receiving the 3HP regimen, those receiving INH/6H (SHR = 4.99, 95% CI: 2.45, 10.07) and those who did not receive TPT (SH = 12.48, 95% CI: 5.47, 28.49) had significantly higher subdistribution hazards of incident TB. Likewise, advanced WHO clinical stage, BMI < 18.5 kg/m^2^ and HBg < 11 g/dL remained independently associated with an increased risk of incident TB after accounting for the competing risk of death (Table 4).

**TABLE 4:** Fine and Gray competing risks regression analysis for predictors of incident tuberculosis among HIV‐positive adults on ART at the Public Health Facility in southern Ethiopia from 1 October 2020 to 31 October 2025.

Variables	SHR	95% CI	*p* value
TPT regimen received			
3HP	1	1	1
INH/6H	4.97	2.45–10.08	< 0.001^∗^
Not received TPT	12.49	5.47–28.50	< 0.001^∗^
WHO clinical stage			
I	1	1	1
II	3.39	1.57–7.33	0.002^∗^
III	5.43	2.65–11.11	< 0.001^∗^
IV	7.52	3.42–16.55	< 0.001^∗^
Functional status			
Working	1	1	1
Nonworking	1.26	0.78–2.03	0.352
BMI			
< 18.5	4.36	2.24–8.46	< 0.001^∗^
≥ 18.5	1	1	1
Patient haemoglobin levels			
< 11	2.49	1.39–4.44	0.002^∗^
≥ 11	1		1
CD4			
< 200	1.71	0.81–2.03	0.16
≥ 200	1		1
Adherence to ART			
Good	0.44	0.17–1.08	0.07
Fair	0.77	0.29–2.027	0.59
Poor	1	1	1

^∗^Statistically significant.

Based on the Kaplan–Meier survival curves, participants who received the 3HP regimen demonstrated the highest cumulative survival probability throughout the 60‐month follow‐up (96%), followed by the INH/6H group (57%), while those who did not receive any TPT consistently exhibited the lowest survival rates (49%). The curves for three groups diverged early and remained distinctly separated over time, indicating a graded survival benefit associated with TPT receipt (S2: Supporting figure 1).

Smoothed hazard functions were consistent with the multivariable Cox regression findings. PLHIV who did not receive TPT experienced the highest hazard of incident TB during follow‐up, followed by those receiving INH/6H, whereas PLHIV receiving the 3HP regimen consistently had the lowest hazard. The smoothed hazard function curves visually support the multivariable findings, which showed that relative to the 3HP reference group, the hazard of TB was significantly higher among participants who received INH/6H and among those who did not receive any TPT (Figure 4).

**FIGURE 4:**
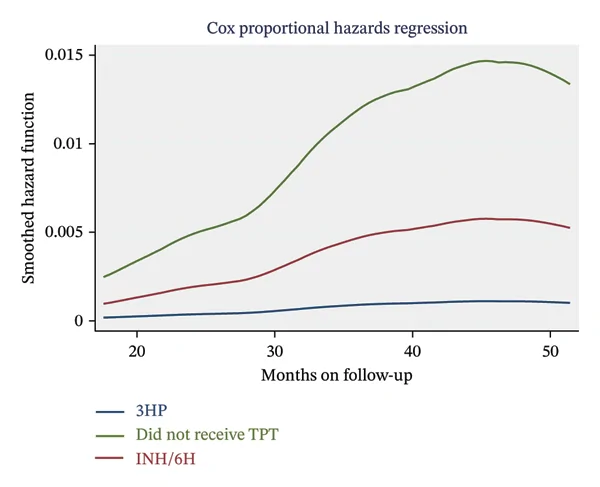
Smoothed hazard functions for incident tuberculosis by TB preventive regimen among people living with HIV receiving ART in public hospitals of West Arsi Zone, Oromia Region, south‐eastern Ethiopia, from 1 October 2020 to 31 October 2025.

Kaplan–Meier curves showed that participants with a value ≥ 11 consistently maintained higher cumulative survival probabilities than those with a value < 11 over the entire 60‐month follow‐up period. The survival curves progressively diverged from early follow‐up onward. The survival curves progressively diverged from early follow‐up onward (S2: Supporting figure 3).

The smoothed hazard functions showed significantly lower TB incidence among patients with HBg ≥ 11 g/dL compared to patients with HBg < 11 g/dL. The separation between the two curves was maintained without crossing, suggesting that the relative effect of HBg on TB risk remained stable over time (Figure 5).

**FIGURE 5:**
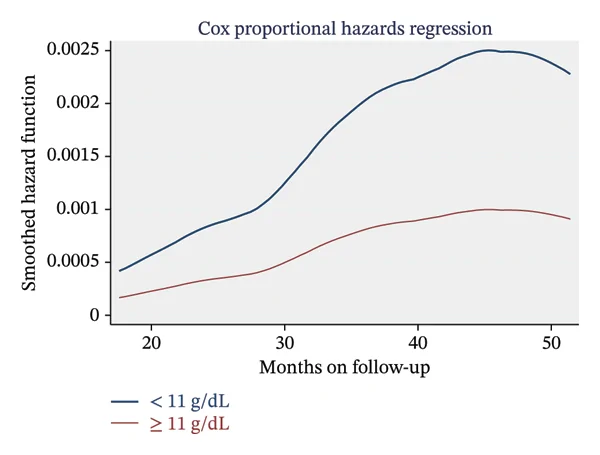
Smoothed hazard functions for incident tuberculosis by haemoglobin levels among people living with HIV receiving ART in public hospitals of West Arsi Zone, Oromia Region, south‐eastern Ethiopia, from 1 October 2020 to 31 October 2025.

Kaplan–Meier survival plot revealed a clear inverse relationship between advanced WHO clinical stage and cumulative survival, with participants in Stage I demonstrating the highest survival probabilities and those in Stage IV consistently showing the lowest throughout the 60‐month follow‐up period. The four survival curves separated distinctly from early follow‐up and remained stratified overtime (S2: Supporting figure 5).

The smoothed hazard functions indicated that HIV‐positive individuals classified as having WHO Clinical Stages II, III and IV diseases were at a considerably higher risk of developing TB than those with WHO Clinical Stage I disease. Across both groups, advanced stages (III/IV) consistently show a higher event rate than early stages. For Stages I and II, the second group demonstrated a clear protective effect, with notably lower cumulative incidence (Figure 6).

**FIGURE 6:**
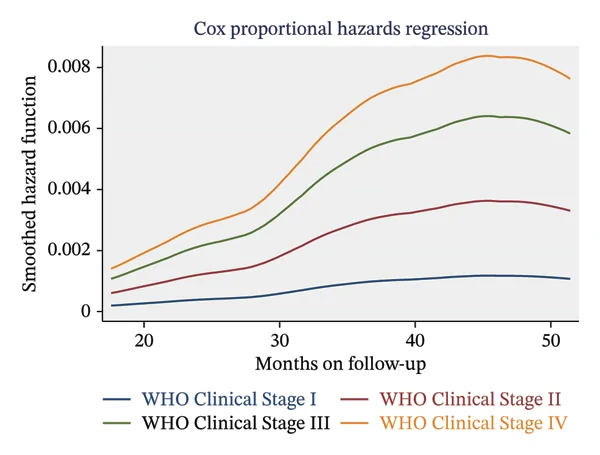
Smoothed hazard functions for incident tuberculosis by WHO clinical stage among people living with HIV receiving ART in public hospitals of West Arsi Zone, Oromia Region, south‐eastern Ethiopia, from 1 October 2020 to 31 October 2025.

Kaplan–Meier survival estimates demonstrated that participants with a BMI of ≥ 18.5 consistently maintained higher cumulative survival probabilities (94%) throughout the follow‐up period compared to those with a BMI < 18.5 (57%) (S2: Supporting figure 7).

The smoothed hazard functions demonstrated, compared with those with a BMI ≥ 18.5 kg/m^2^, that HIV‐positive individuals with a BMI < 18.5 kg/m^2^ were at greater risk. Over the follow‐up period, the cumulative incidence of the outcome remained at 0.00 throughout in the BMI ≥ 18.5 kg/m^2^ group. In contrast, among participants with BMI < 18.5, the incidence rose steadily from 0.01 at 20 months to 0.02, 0.05 and 0.06 at 30, 40 and 50 months, respectively. This suggests that the lower BMI was linked to increased risk of TB over time, with curves diverging notably after 30 months (Figure 7).

**FIGURE 7:**
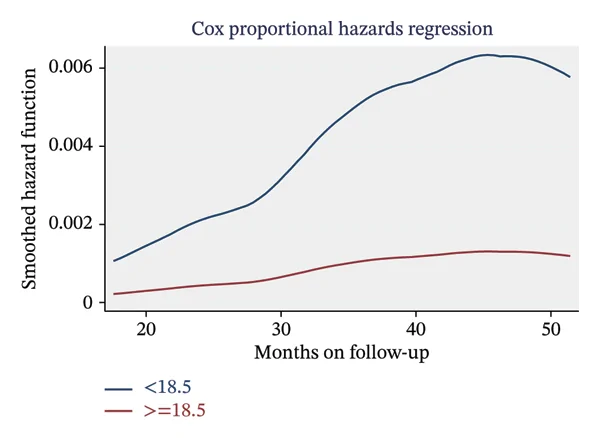
Smoothed hazard functions for incident tuberculosis by body mass index (BMI) among people living with HIV receiving ART in public hospitals of West Arsi Zone, Oromia Region, south‐eastern Ethiopia, from 1 October 2020 to 31 October 2025.

## 4. Discussion

This study investigated the incidence of TB and its predictors among HIV‐positive adults on ART in West Arsi Zone, Ethiopia. This study evaluated the incidence of TB among adults living with HIV receiving ART in West Arsi Zone, Ethiopia. A total of 426 adults living with HIV were enrolled in the study, contributing a cumulative 1867 PYs of follow‐up. During this period, 71 patients (16.7%) developed TB, with an overall incidence density of 3.78 cases of TB per 100 PYs.

The independent risk factors significantly associated with TB among HIV‐positive patients were advanced WHO clinical stages, BMI < 18.5 kg/m^2^, HBg level < 11 g/dL and receiving INH/6H and not receiving TPT. Patients in advanced stages of HIV (as classified by the WHO) were more likely to develop TB. This suggests that individuals with more severe HIV‐related immunosuppression may be at greater risk, likely due to their immunosuppression. A BMI < 18.5 kg/m^2^ was an independent predictor of incident TB. This finding is biologically plausible, as undernutrition or malnutrition compromises immune function, thereby increasing susceptibility to TB infection and disease progression.

To evaluate whether death influenced the estimated associations, we performed a Fine and Gray competing risks regression analysis treating death as a competing event. The competing risk analysis yielded findings consistent with those from the cause‐specific Cox regression hazards model, indicating that advanced WHO clinical stage, low BMI, low HBg level, receiving INH/6H and not receiving TPT remained significant predictors of incident TB. These consistent findings suggest that the primary conclusions of the study are robust and unlikely to be materially affected by the competing risk of death.

The TB incidence density observed in this study (3.78 per 100 PYs) was comparable to the pooled incidence rate reported in a recent systematic review and meta‐analysis of HIV‐positive individuals in sub‐Saharan Africa (3.49 per 100 PYs) [21] and a systematic review from Ethiopia (4.8 per 100 PYs) [22] and lower than incidence rates reported from north‐east Ethiopia (8.6 per 100 PYs) [10] and Addis Ababa (6.82 per 100 PYs) [13], lower incidence rates reported in Denmark at 2.9% [23]. This difference may reflect variation in study period, implementation of TPT (particularly the introduction of the 3HP regimen), ART coverage, baseline clinical characteristics of participants, duration of follow‐up and the intensity of TB screening and HIV care across settings. TB observed (16.7%) among the HIV‐positive patients in this study is similar to findings from research conducted in Debre Markos, Ethiopia (16.9%) [8], and Tanzania (16.7%) [24]. This indicates that the incidence of TB remains relatively high, highlighting the significant risk of TB within this specific population, regardless of the government’s efforts to implement various initiatives, including TPT with isoniazid–rifapentine (3HP). Additionally, the high burden of HIV in Ethiopia likely heightens susceptibility to TB, highlighting a pervasive health issue that contributes to consistent findings across different studies. Furthermore, lower cumulative incidence has been reported in several locations, including Saudi Arabia at 9.5% [25], in Addis Ababa, Ethiopia, where the incidence was reported at 14% [14], and Debra Markos at 12.6% [9]. In contrast, higher incidence rates have been reported in north‐east Ethiopia, with an incidence of 26.3% [26], Addis Ababa at 25.8% [13], Jimma at 17.3% [16], Hawassa at 29.06% [27] and Arba Minch at 21.4% [17]. This discrepancy may be partly explained by variations in study periods and the timing of national policy implementation. Notably, earlier studies were conducted before the nationwide implementation of the 3HP regimen and focused on socioeconomic status, access to health services, and environmental factors in the populations being studied, which indicates that TB incidence rates are influenced by both local factors and policy‐related variables. This highlights the importance of generating local evidence to inform both national policy and global strategies aimed at ending the TB epidemic among PLHIV.

In the present study, compared to the 3HP regimen, HIV‐positive individuals who received 6‐month IPT (INH/6H) had a fivefold higher hazard of developing TB, while those who did not receive any TPT had a more than 12‐fold higher hazard of incident TB (Figure 4). Although direct comparative studies of TB incidence between 3HP and 6H among PLHIV are limited in Ethiopia, the national scale‐up of 3HP has substantially improved overall TPT uptake among this population [28, 29]. Our findings are consistent with recent programmatic evidence from Kenya [19] and Pakistan [20], which reported higher uptake and completion rates with 3HP than with 6H, resulting in lower TB incidence among individuals who completed preventive therapy. Similarly, a network meta‐analysis across 14 countries reported higher treatment completion rates with 3HP than with longer isoniazid regimens [30].

However, our findings differ from those of a network meta‐analysis [31], which reported no significant difference in TB incidence between rifamycin‐containing regimens, including 3HP, and 6–12 months of isoniazid (incidence rate ratio ≈ 1.0). Likewise, another systematic review concluded that 3HP was noninferior, rather than superior, to longer isoniazid regimens in preventing active TB (RR = 0.73, with overlapping confidence intervals) [32]. These discrepancies may be explained by differences in study design and implementation. The meta‐analyses pooled data from randomised controlled trials or directly observed therapy (DOT), while our study measured effectiveness in a routine programmatic setting.

In our study, HIV‐positive patients with a low BMI were nearly 4.4 times more likely to develop TB than their counterparts (Figure 7), which aligns with findings from various regions of Ethiopia, including Gojjam in Northwest [33], Northeast [10] and Addis Ababa [14], as well as research in Tanzania [24]. Furthermore, a meta‐analysis in sub‐Saharan Africa reported that underweight HIV‐positive individuals were significantly more likely to develop TB than were those with a normal body weight. The possible reason could be that low body weight reflects social factors such as poverty, limited access to healthcare, inadequate nutrition and poor living conditions, all of which can contribute to a higher risk of TB. Furthermore, these factors may contribute to impaired immune function, reducing the body’s ability to mount an effective immune response and making individuals more susceptible to opportunistic infections, such as TB. This association highlights underlying issues of poverty and inadequate access to nutrition, underscoring the need for routine nutritional screening among all PLHIV, along with targeted nutritional support for those who are underweight.

The present study revealed that the baseline WHO clinical stage; specifically, patients in WHO Clinical Stages IV, III and II were approximately 7.52, 5.42 and 3.44 times, respectively, more likely to develop TB than those starting follow‐up at the first stage, WHO Clinical Stage I (Figure 6). These findings align with those of previous studies conducted in various regions of Ethiopia, including Arba Minch [17], Northeast Ethiopia [10], Jimma [16], Hawassa [22], Debra Markos in Northwest Ethiopia [8] and Harar [34]. Furthermore, a meta‐analysis encompassing studies from Ethiopia [22, 26], Tanzania [24], Addis Ababa [14] and other areas in sub‐Saharan Africa [21] further supports these findings. A possible explanation might be that patients in higher WHO clinical stages have more advanced HIV infections, leading to a greater degree of immunosuppression, which increases susceptibility to opportunistic infections such as TB. Additionally, individuals in later clinical stages may have higher viral loads and more severe manifestations of HIV and experience additional comorbidities, which can accelerate the progression of TB. This implies that healthcare providers and clinicians should prioritise early diagnosis and treatment of HIV to prevent progression to advanced clinical stages through integrated HIV and TB care programmes that emphasise regular monitoring and timely initiation of ART.

We also found that HIV‐positive patients with HBg levels < 11 g/dL had a 2.5‐fold higher hazard of developing TB compared to those with HBg ≥ 11 g/dL. This finding is consistent with a prior study conducted in Debra Markos, Ethiopia, which reported that patients with low HBg levels had a 3.5‐fold higher hazard of developing TB (AHR 3.5, 95% CI: 2.1, 5.8) [8] (Figure 5). Similarly, a systematic review and meta‐analysis of Ethiopian cohort studies found that anaemic HIV‐infected persons had a pooled 2.3‐fold higher risk of developing TB compared with nonanaemic patients (HR = 2.30, 95% CI: 1.75, 3.02) [22]. This is strikingly similar to the effect observed in the current study (AHR = 2.46), supporting the validity of the current finding. The consistency between our findings and other studies could be attributed to biological mechanisms such as anaemia, which is a marker of advanced HIV disease, and increases susceptibility to reactivation of latent TB or progression after new infection, and low HBg frequently coexists with malnutrition, advanced WHO clinical stage and low CD4, increasing the risk of active TB.

### 4.1. Limitations

Although we made significant efforts to identify predictors of TB incidence among HIV‐positive individuals using 5 years of retrospective data, we acknowledge limitations when interpreting the findings. One major constraint is that conducting research in a single zone of Ethiopia may limit the generalisability of the results to other regions of the country with different population characteristics or healthcare contexts. Additionally, owing to the reliance on existing medical records, any variables not originally recorded could not be included in the analysis. Although no randomly selected patient records were excluded because of missing data, this study was based on routinely collected retrospective medical records. The findings may still be subject to limitations inherent to retrospective data, including the possibility of recording errors, residual confounding and potential selection bias related to the availability and quality of clinical documentation. The final parsimonious multivariable Cox regression model was based on recommended EPV considerations, supported by the 71 incident TB events observed. Even though the sample size was initially determined using a proportion‐based approach, the number of events was adequate for the final model.

## 5. Conclusion

This study highlights that TB remains a significant comorbidity among HIV‐positive patients, with an incidence density of 3.78 cases per 100 PYs, underscoring the persistent burden of TB in this population despite preventive efforts. Advanced WHO clinical stages, undernutrition (BMI < 18.5 kg/m^2^), anaemia (HBg < 11 g/dL), receipt of INH/6H and not receiving any TPT were independent predictors of TB among HIV‐positive individuals.

Our findings suggest that nutritional screening and support may reduce TB risk, and policymakers may wish to consider strengthening community‐based intervention strategies. Additionally, future research should encompass thorough studies that integrate qualitative methods; examine the pooled incidence of TB following the initiation of 3HP; assess trends in LTBI after TPT, particularly post‐3HP initiation; and explore the knowledge, attitudes and practices of both HIV‐infected patients and healthcare providers related to TPT, as well as the overall implementation status of TPT.

Finally, policymakers must strengthen health system capacity for community‐based TB prevention strategies, ensuring accessibility to preventive therapies and fostering public awareness of the risks associated with TB–HIV coinfection to effectively combat this dual health challenge for this vulnerable population. In addition, the results obtained from this study will be helpful for healthcare professionals involved in the TB/HIV prevention and control unit of the West Arsi HF. Additionally, the study provides insights for further intervention studies.

**Nomenclature**
AIDS: Acquired immune deficiency syndrome
AHR: Adjusted hazard ratio
CI: Confidence interval
FMOH: Federal Ministry of Health
HIV: Human immunodeficiency virus
HR: Hazard ratio
SPSS: Statistical Package for Social Science
SHR: Subdistribution hazard ratio
TB: Tuberculosis
TPT: Tuberculosis preventive therapy
WHO: World Health Organization

## Author Contributions

Edao Sinba Etu and Edao Wajiso Kallo led all the research activities, including conceptualisation, data curation, data analysis, investigation, methodology, project administration, software, visualisation and writing–original draft preparation. Musa Kumbi Ketero and Habtemu Jarso Hebo contributed to the data analysis, methodology and software. Kelil Husen Lenbo, Shumi Negewo Berarti, Musa Kumbi Ketero and Habtemu Jarso Hebo contributed to the methodology, project administration and supervision. Biftu Geda contributed to the software, supervision and methodology. Kemal Ahmed Kuti contributed to the software, supervision and methodology. MS contributed to the software, supervision and methodology.

## Funding

This research did not receive any specific grant from funding agencies in the public, commercial or not‐for‐profit industries. Madda Walabu University supported the data collection.

## Disclosure

All the authors reviewed and approved the final manuscript.

## Ethics Statement

The ethical review committee of Madda Walabu University College of Health Science granted ethical clearance before the commencement of data collection (Ref. No. IRERC/046/2023). Additionally, a permission letter was obtained from the Oromia Regional Health Bureau ethical review team and West Arsi Health Office. Permission to access patients’ records was obtained from all participating health facilities after the study’s purpose, and anticipated benefits were explained to their administrators and staff. To ensure confidentiality, we utilised a unique study identification number throughout the process, and no patient identifiers were registered in the extraction tool. Patient consent was not required for this study because of its retrospective nature (not applicable).

## Consent

The authors have nothing to report.

## Conflicts of Interest

The authors declare no conflicts of interest.

## Supporting Information

Additional supporting information can be found online in the Supporting Information section.

## Supporting information


**Supporting Information** Additional file S1: Appendix A and B: Institutional permission and Record review checklist (English version). S2: Kaplan–Meier estimate plots, log‐minus‐log plots, Cox–Snell residual plot and table for sample size calculated for the second specific objective.

## Data Availability

The dataset generated and analysed during the current study is available in the figshare repository under the file name shared file, https://figshare.com/s/77bd6c4be3c7685e1408.
