# Supplementary material for: Tuberculosis Incidence and Predictors Among People Living With HIV After Isoniazid–Rifapentine (3HP) Preventive Therapy in Ethiopia: A Retrospective Cohort Study
Source: J Trop Med. 2026 Oct 6;2026:4521687. doi: 10.1155/jotm/4521687 (PMC13640529; doi:10.1155/jotm/4521687)
Supplement: Supplementary file 1 [file JOTM-2026-4521687-s001.docx]

## S1: Annex A: English Institutional permission

**NB: This *form was used to obtain institution permission from the participating health facilities rather than individual patient consent***

Good morning (Good afternoon/evening). My name is --------------------, I am working as data collector/ record reviewer for MWU University Shashemene campus PhD student’s mega project entitled as ‘’ Universal Test and Treat strategy of HIV: outcomes, co-morbidities, and implications for intervention sub theme Management of Latent Tuberculosis Infection Treatment among people living with HIV/AIDS in West Arsi: Incidence, Adherence, Trend, Implementation status, treatment Outcome and nutritional status.’’ The purpose of this study is to generate information which may help health services providers, NGOs and others working in this area to take actions based on the findings. I would like to request your office’s permission to conduct this study, which involves the assessment of ART medical records.

**Confidentiality**: The information in your institutional medical record is confidential. There was no information that would identify patient person identify. The study's conclusions were generic for the study community and would not reflect anything particular about individual person. The questionnaire was coded to exclude showing the names. No oral or written reports included any references that could link participants to the research.

Principal Investigator (PI): Edao Sinba

Mobile phone: +251-912008000 Email: [esetu912008000@ymail.com](mailto:esetu912008000@ymail.com)

## Annex B: Record review checklist English version

Name of data collector---------------------------Signature------------Date ----------------
Name of supervisor--------------------------------Signature--------------Date --------------
Questionnaire code------------------------health center/hospital-------------------Kebele

Part one Socio-Economic and Demographic Characteristics

| S.No | Item | Categories | Skip |
| --- | --- | --- | --- |
| 101 | Facility name |  |  |
| 102 | MRN |  |  |
| 103 | Unique ART no |  |  |
| 104 | Enrolment Date |  |  |
| 105 | Unit TB No |  |  |
| 106 | Age in completed years | ----------years |  |
| 107 | Sex | 1)Male  2)Female |  |
| 108 | Religion | 1)Protestant  2)Orthodox  3)Muslim  4)other |  |
| 109 | Residency | 1.Urban  2.Rural |  |
| 110 | Marital status | 1)Single  2)Married  3)Widowed  4) Divorced |  |
| 111 | Educational status | 1)Unable to read and write  2)Grade 1-6  3)Grade 7-12  4) Diploma and above |  |
| 112 | Occupation? | 1)Farmer  2)merchant  3)Daily labor  4)Other (specify) |  |
| 113 | Family size | $1)\leq$5  2) >5 |  |

Part two Clinical profile

| 114 | | Do you have TB treatment history during follow up? | Yes  No |  |
| --- | --- | --- | --- | --- |
| 115 | | If yes for Q 115 what is type of TB? | Pulmonary TB  Extra pulmonary TB  Other |  |
| 116 | | WHO clinical stage | 1) I 2) II 3) III 4) IV |  |
| 117 | | Duration on ART (month) | ------months |  |
| 118 | | Functional status | 1) Working 2) Ambulatory 3) Bed ridden |  |
| 119 | | What was BMI? | -----kg/m2 |  |
| 120 | | Nutritional status | Normal  Moderate wasting  Severe wasting |  |
| 121 | | HB level in g/dL | -------g/dl |  |
| 122 | Months of follow up | | Months |  |
| 123 | Does TB preventive therapy was taken? | | Yes  No |  |
| 1124 | Type of TPT received | | 1. 3HP 2. IPT 3. Not receive any TPT |  |
| 125 | Does Cotrimoxazole preventive therapy taken? | | Yes  No |  |
| 126 | VL | | 1.suppressed  2.unsuppressed  3.not done |  |
| 127 | Does regimen changed during follow up ? | | Yes (reason)  No |  |
| 128 | If yes Q ,reason for switch first regimen | | Side effect  Pregnancy  Tuberculosis  Others |  |
| 129 | Disclosure status | | Disclosure  Not disclosure |  |
| 130 | CD4 cell count | | ---------cells/mm2 |  |
| 131 | ARV/adherence | | Good  Fair  Poor |  |
| 132 | status | | Lost/TO/died |  |

**S2: Kaplan-Meier estimate plots, log-minus- log plots, Cox-Snell residual plot and table for sample size calculated for second specific objectives**

Based on the Kaplan-Meier survival curves, participants who received the 3HP regimen demonstrated the highest cumulative survival probability throughout the 60-month follow-up (96%), followed by the INH/6H group (57%), while those who did not receive any TPT consistently exhibited the lowest survival rates (49%). The curves for three groups diverged early and remained distinctly separated over time, indicating a graded survival benefit associated with TPT receipt **(Suppl. Figure 1)**.

The Kaplan-Meier curves showed that participants receiving 3HP maintained the highest TB-free survival throughout follow-up. The TB-free survival for the INH/6H and non-TPT groups overlapped during part of follow-up period, suggesting similar unadjusted survival probabilities early in follow-up. However, the no-TPT group demonstrated a lower TB-free survival toward the end of follow-up. In the adjusted Cox proportional hazards model, participants who did not receive TPT had a substantially higher hazard of developing TB than those receiving INH/6H, indicating that adjustment for prognostic factors strengthen the observed association.

Supplementary Figure 1: Kaplan-Meier survival plot for incident tuberculosis by TB preventive regimen among people living with HIV receiving ART in public hospitals of West Arsi Zone, Oromia Region, south-eastern Ethiopia, from October 1, 2020, to October 31, 2025

**Supplementary Figure 2**: Log-minus-log survival curves demonstrating the proportional hazards assumption across TPT regimen groups among people living with HIV receiving ART in public hospitals of West Arsi Zone, Oromia Region, south-eastern Ethiopia, from October 1, 2020, to October 31, 2025

Kaplan-Meier curves showed that participants with a value ≥11 consistently maintained higher cumulative survival probabilities than those with a value <11 over the entire 60- month follow-up period. The survival curves progressively diverged from early follow-up onward. The survival curves progressively diverged from early follow‑up onward **(Suppl. Figure 3)**.

**Supplementary Figure 3:** Kaplan–Meier survival plots by type of Haemoglobin level among people living with HIV receiving ART in public hospitals of West Arsi Zone, Oromia Region, south-eastern Ethiopia, from October 1, 2020, to October 31, 2025

**Supplementary Figure 4:** Log-minus-log survival curves demonstrating the proportional hazards assumption across Haemoglobin level groups among people living with HIV receiving ART in public hospitals of West Arsi Zone, Oromia Region, south-eastern Ethiopia, from October 1, 2020, to October 31, 2025

Kaplan-Meier Survival plot revealed a clear inverse relationship between advanced WHO clinical stage and cumulative survival, with participants in Stage I demonstrating the highest survival probabilities and those in Stage IV consistently showing the lowest throughout the 60-month follow-up period. The four survival curves separated distinctly from early follow-up and remained stratified overtime **(Suppl. Figure 5)**.

**Supplementary Figure 5:** Kaplan‒Meier plots of the incidence of TB according to the WHO clinical stage among people living with HIV receiving ART in West Arsi Zone public hospitals in Oromia, southeastern Ethiopia, from October 1, 2020, to October 31, 2025

**Supplementary Figure 6:** Log-minus-log survival curves demonstrating the proportional hazards assumption across WHO clinical stage groups among people living with HIV receiving ART in public hospitals of West Arsi Zone, Oromia Region, south-eastern Ethiopia, from October 1, 2020, to October 31, 2025

Kaplan- Meier Survival estimates demonstrated that participants with a BMI of ≥18.5 consistently maintained higher cumulative survival probabilities (94%) throughout the follow-up period compared to those with a BMI<18.5(57%) **(Suppl. Figure 7).**

Supplementary Figure 7: Kaplan‒Meier risk plots based on the BMI among people living with HIV receiving ART in West Arsi Zone public hospitals in Oromia, southeastern Ethiopia, from October 1, 2020, to October 31, 2025

**Supplementary Figure 8:** Log-minus-log survival curves demonstrating the proportional hazards assumption across BMI groups among people living with HIV receiving ART in public hospitals of West Arsi Zone, Oromia Region, south-eastern Ethiopia, from October 1, 2020, to October 31, 2025

**Supplementary Figure 9**: Cox Snell residual plot for predictors

**Supplementary Table 1: Sample size calculated for second specific objectives**

| Variables | | CI% | Power% | OR | Ratio | Percent in unexposed | Lost to follow up | Sample size | Ref |
| --- | --- | --- | --- | --- | --- | --- | --- | --- | --- |
| WHO stage | III | 95 | 80 | 20.26 | 1 | 6.08 | 10% | 35 | (Dalbo and Tamiso, 2016) |
|  | IV | 95 | 80 | 22.9 | 1 | 6.08 | 10% | 57 | “ |
| smoking status | | 95 | 80 | 2.82 | 1 | 18.98 | 10% | 185 | “ |
| Hemoglobin | | 95 | 80 | 2.56 | 1 | 18.38 | 10% | 231 | “ |
